# Unveiling the Potential of Bioinoculants and Nanoparticles in Sustainable Agriculture for Enhanced Plant Growth and Food Security

**DOI:** 10.1155/2023/6911851

**Published:** 2023-11-29

**Authors:** Arun Karnwal, Aradhana Dohroo, Tabarak Malik

**Affiliations:** ^1^Department of Microbiology, School of Bioengineering & Biosciences, Lovely Professional University, Phagwara, Punjab 144411, India; ^2^Baddi University of Emerging Sciences and Technologies, Baddi, Himachal Pradesh 173405, India; ^3^Department of Biomedical Sciences, Institute of Health, Jimma University, Ethiopia

## Abstract

The increasing public concern over the negative impacts of chemical fertilizers and pesticides on food security and sustainability has led to exploring innovative methods that offer both environmental and agricultural benefits. One such innovative approach is using plant-growth-promoting bioinoculants that involve bacteria, fungi, and algae. These living microorganisms are applied to soil, seeds, or plant surfaces and can enhance plant development by increasing nutrient availability and defense against plant pathogens. However, the application of biofertilizers in the field faced many challenges and required conjunction with innovative delivering approaches. Nanotechnology has gained significant attention in recent years due to its numerous applications in various fields, such as medicine, drug development, catalysis, energy, and materials. Nanoparticles with small sizes and large surface areas (1-100 nm) have numerous potential functions. In sustainable agriculture, the development of nanochemicals has shown promise as agents for plant growth, fertilizers, and pesticides. The use of nanomaterials is being considered as a solution to control plant pests, including insects, fungi, and weeds. In the food industry, nanoparticles are used as antimicrobial agents in food packaging, with silver nanomaterials being particularly interesting. However, many nanoparticles (Ag, Fe, Cu, Si, Al, Zn, ZnO, TiO_2_, CeO_2_, Al_2_O_3_, and carbon nanotubes) have been reported to negatively affect plant growth. This review focuses on the effects of nanoparticles on beneficial plant bacteria and their ability to promote plant growth. Implementing novel sustainable strategies in agriculture, biofertilizers, and nanoparticles could be a promising solution to achieve sustainable food production while reducing the negative environmental impacts.

## 1. Introduction

**Figure 1:**
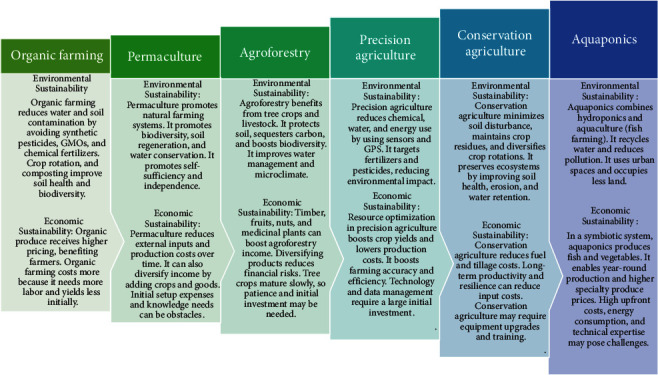
Environmental and economic sustainability features of different types of sustainable agricultural practices.

Population increase and industrial growth have resulted in a rise in food consumption and the utilization of chemical fertilizers on a higher level to fulfill this need. However, the widespread use of agrochemicals to improve agricultural output has destroyed soil microorganism communities, contaminated groundwater and soil, damaged biodiversity, and impacted ecosystem balance [1–3]. Finding adequate replacements to replace or significantly reduce chemical inputs in agriculture has never been more crucial amid the severe environmental impact of chemicals and the limitation of land resources [3]. Sustainable agricultural practices are crucial for the long-term health of our planet and the well-being of future generations. These practices are aimed at minimizing the environmental impact of agriculture while ensuring food security and promoting economic viability. There are several different types of sustainable agricultural practices, i.e., organic farming, permaculture, agroforestry, precision agriculture, conservation agriculture, and aquaponics, that contribute to these goals as mentioned in Figure 1. By adopting these and other sustainable agricultural practices, we can protect natural resources, mitigate climate change, promote biodiversity, and ensure the availability of nutritious food for generations to come. The integration of these practices with innovative technologies (i.e., bioinoculants and nanotechnology) and the support of policymakers, farmers, and consumers are essential for building a sustainable and resilient agricultural system.

Sustainable agriculture requires balancing agricultural and environmental challenges. Therefore, it is possible to obtain high agriculture production with minimal environmental damage by efficiently utilizing biofertilizers, biopesticides, ecologically sensitive water usage, and soil management [4, 5]. Utilizing bacteria that promote plant growth, commonly called PGPB (plant-growth-promoting bacteria), is an eco-friendly and advantageous approach to achieve these objectives. These free-living PGPBs live in a mutually beneficial relationship with the plant's roots (rhizospheric/exogenous PGPB), whereas endogenous PGPBs live in the flowers, leaves, or other plant parts that are not visible to the naked eye [5–7]. Both exogenous and endogenous PGPBs boost plant growth through several approaches, like improved nutrient uptake, stress tolerance, and defense against phytopathogens [8]. Therefore, microorganism-based biofertilizers have progressively demonstrated economic promise for application in sustainable agriculture. The global market for plant biofertilizers is anticipated to increase by 12% annually and reach $6 million by 2024 [8, 9].

However, numerous destabilizing causes and irregularity of microbial inoculants in the field impede biofertilizer effectiveness [9]. Nanotechnology has been actively utilized in intelligent farming in the past years [10]. Nanoparticles (NPs) are being explored for their possible application in overcoming issues linked with biofertilizers, such as sensitivity to temperature change and dehydration, storage stability, and reproducibility, because of their small sizes and distinctive characteristics relative to their bulk materials [10–12]. It has been suggested that nanostructures, also known as nanobiofertilizers, can boost the capability of PGPB for use as inoculants. Gold, titanium, zinc, and silicone nanoparticles have been shown to enhance the beneficial qualities of PGPB in plants and raise the number of microbial cells [13]. Nanomaterials are widely regarded as the next technical and scientific leap in helping human advancement. From this perspective, using nanotechnology to benefit microbes is a possible strategy for improving agricultural, financial, and environmental benefits [10]. Therefore, the objective of this review is to present a summary of the benefits that may be achieved by formulations containing inorganic NPs linked with PGPB for plant growth.

## 2. Sustainable Agriculture Using Nanotechnology

Nanotechnology comprises the application of substances with unique features. These features might arise from either the differential geometry of nanoparticles, the nanoscale level results in unique and distinct quantum confinement effects, or the availability of highly reactive surfaces, which are only found at the nanoscale level [14]. Because of the smaller size, increased surface area-to-weight ratio, and material attributes contrasted to the macroscopic level, nanomaterials offer significant reactivity and enhanced bioavailability/bioactivity, adhesion, and surface effects, among other benefits [15]:

- *Enhanced reactivity*: nanomaterials possess a high surface-to-volume ratio, which increases their reactivity compared to bulk materials [11]. This enhanced reactivity allows for improved performance in various agricultural processes. For example, nanoscale catalysts can facilitate more efficient chemical reactions, such as the degradation of pesticides or the conversion of nutrients into plant-available forms [7]
- *Bioavailability/bioactivity*: nanomaterials can enhance the bioavailability and bioactivity of agricultural compounds, such as fertilizers, pesticides, and plant growth regulators [16]. The surface area available for interaction with living organisms is increased by reducing the particle size to the nanoscale. This can enhance nutrient absorption by plants, improve pesticide uptake by pests, and increase the effectiveness of growth-promoting substances [12, 17]
- *Adhesion*: nanomaterials can improve the adhesion properties of agricultural formulations, leading to better performance and reduced losses [18]. For example, nanoparticles can be used as adhesion promoters in pesticides or foliar fertilizers, enabling them to stick better to plant surfaces. This enhances their efficacy, reduces runoff, and minimizes environmental impact [19]
- *Surface effects*: nanomaterials can modify the surface properties of agricultural substrates, resulting in beneficial effects. Surface modification with nanoparticles can enhance water repellency or retention, improve soil structure, and increase nutrient retention in the root zone [16]. These surface effects can contribute to enhanced plant growth, reduced water usage, and improved soil health

NPs can be produced by a single metal (like gold) or by a combination of materials, like those in oxides (TiO_2_, SiO_2_, and ZnO). To maintain a sustainable agricultural system, innovative solutions are becoming increasingly required. Further, nanotechnology-based strategies are effective tools for overcoming obstacles in the food and agriculture sectors, like the increasing need for food, food security, crop diseases, and environmental degradation [20]. Scientists have recently begun exploring nanomaterials with minimal adverse ecological impact to boost agricultural yield. Nanostructures are utilized in various fields within the agricultural industry, like seed science, nanofertilizers, nanoherbicides, water resource management, nanoscale transporters, biosensors, agriculture engineering, and zoology [11, 21]. For instance, nanomaterial-based intelligent agriculture systems provide efficient absorption of nutrients by plants, distribution, and regulated discharge of chemicals in targeted areas, prompt disease detection, and environmental protection [11]. To enhance targeted delivery and release in plants at relatively low dosages per application, nanostructured materials have been employed in conjunction with agriculture products (nanofertilizers and nanopesticides). Due to this, fewer harmful residues are left in the soil to cause long-term damage to the ecosystem [17]. Smart seeds can be loaded with nanoencapsulations containing specific bacterial species to improve seeding rates, assure proper field standing, and enhance crop yield [22]. Smart seeds can also be scattered across a field and programmed to grow under optimal temperature, moisture, and pH conditions.  Table 1 shows the nanoparticle-induced gene expression in plants.

## 3. Biofertilizer Formulations Using Nanomaterials

Plant life depends on various factors in the ecosystem. It is widely established that PGPB promotes plant development through various direct and indirect processes. Direct processes comprise the absorption of vital minerals from the soil, like nitrogen, phosphorus, and iron, and the production or regulation of plant hormones, including gibberellin, cytokinin, and auxin [8, 9]. Indirectly, PGPB supports plants in withstanding biotic stresses through the production of antimicrobials, peroxidase, and related substances; the regulation of reactive oxygen species; the reduction of nutritional supply of pathogens; the synthesis of pathogen-inhibiting VOCs; and the promotion of ISR (induced-systemic-resistance) in the plant [9, 17, 26].

Plants can only absorb 30–50% of synthetic fertilizers; therefore, the remaining amount pollutes the groundwater. Therefore, saturation has led to a decline in fertilizer effectiveness [9]. Biofertilizers are made up of active or dormant microorganisms (inoculants) in a composition that allows for convenient usage and long-term storage, functioning as a microbe distribution tool to boost plant nutrient supply [9]. Potassium solubilizers, phosphorus solubilizers, nitrogen fixers, and biocontrol agents are all types of PGPB employed as biostimulants in agriculture across the globe. N_2_-fixing microbes lead the worldwide biofertilizer industry since plants cannot transform air nitrogen into usable nitrogen [8]. *Pseudomonas*, *Azospirillum*, *Acetobacter*, and *Azotobacter* are the genera that contain most of the species that are utilized as inoculants [18].

Furthermore, *Bacillus* and *Pseudomonas* species are effective plant growth stimulators and biological control agents under stress conditions [5]. Biofertilizers provide several benefits against synthetic fertilizers, including higher sustainability, lower ecological damage, better soil quality, and more affordability for small and marginal farmers. As a result, microbial biofertilizers have the potential to lessen or eliminate the synthetic fertilizer application in agricultural settings, thus mitigating the associated adverse effects [5, 6]. Although PGPBs have potential and are currently utilized commercially as bioinoculants in farming, their proper implementation may not match their anticipated plant productivity due to their soil stability, field applications, and administration techniques [9]. In most cases, bacterial populations immediately decrease after being inoculated, reducing bacterial activity in the rhizosphere. This happens because a variety of variables influence bacterial colonization within plants [27]. To avoid this deterioration in the field, PGPB requires either a suitable microenvironment or long-term structural shielding. In addition, a sufficient number of bacterial cells is required to induce a favorable reaction in the host plant to colonize successfully. For instance, the bacteria *Azospirillum brasilense* has to be present at a density of 10^6^–10^7^ cells per plant [28]. PGPBs in agricultural systems require peat or liquid carriers to stabilize and support bacteria throughout transportation and storage. The carrier, derived from inorganic or organic chemicals or synthesized from biomolecules, is the crucial component of the biofertilizer [29]. In order to reduce production costs without sacrificing quality, it is necessary to look into potential replacement materials for bacterial inoculum. For all these goals, nanomaterials may be a contemporary and efficient strategy to improve the persistence and delivery of good microbes in agriculture. Integrating nanoformulations can provide persistent and reproducible biofertilizers by improving their tolerance to dehydration, heat, and ultraviolet radiation [30]. This method entails incorporating microbial cells with organic or inorganic NPs and administering the microorganisms to specific tissues at certain periods under controlled environments [29].

In most cases, the specificities and relationships among microbes and NPs are determined by the negatively charged groups with enormous specific surface areas. Hydrophobic and positive charge areas on the cell surface and solid particles promote adherence among microbes and nanomaterials [29]. This is even though the overall charge is negative. The NPs can either produce hydrophobic domains on the cell membranes of bacteria or attach to preexisting hydrophobic areas on the bacteria, both of which can result in the emergence of aggregation and bonding arrangements [17].

Moreover, particle interactions with bacteria are caused by electrostatic interaction and bacterial surface chemical reactions, i.e., those related to phospholipid membrane exposure. The primary macromolecules associated with NPs include bacterial cell phospholipids, proteins, lipoteichoic acid (LTA), and lipopolysaccharides (LPS) [26]. The size of the molecules influences the mechanism by which NP is transported into bacterial cells, and there is currently no specific framework that can anticipate the interactions between PGPB and NP. Evidence supports the hypothesis that NPs can influence the PGPB-plant system directly and indirectly [5, 7, 8]. In the direct mode, NPs promote growth-promoting properties by increasing nutrient availability in plants, whereas in the indirect mode, NPs activate the PGPB to do a similar function (Table 2) [7].

NPs have several favorable effects on beneficial bacteria, including increased nitrogen fixation and secondary metabolite synthesis [7, 31]. For example, *Pseudomonas chlororaphis*, a plant-growth-promoting bacterium, produced more IAA (indole-3-acetic acid) when exposed to copper oxide NPs and more siderophore when exposed to zinc oxide NPs, most probably due to ion release [32]. *Nitrosomonas europaea* had a stronger ability for nitrogen fixation after exposure to silver NPs, due to a rise in the activation of nitrification-related genes amoA1 and amoC2 [33]. However, studies [7, 34] have shown that NPs can harm positive bacterial traits. This makes it challenging to anticipate NPs' effects on bacteria because those effects depend on several factors. The exact strategies by which nanomaterials alter bacterial physiology are not fully known; however, they may involve ion exchanges, gene expression alterations, and cell membranes. In addition, the partnership of NPs and plant-growth-promoting bacteria (PGPB) can potentially enhance the inhibition of plant pathogens [2, 17, 35]. The upsurge in the bacterial cell counts gives PGPB an advantage against plant pathogens in the competition for various resources.

Furthermore, higher secondary metabolite synthesis and nutrient intake strengthen plants' resistance to plant pathogens. In particular [36], the introduction of silica nanoparticles causes an increase in the plant cell wall thickness and strength in maize (Table 3). It provides an additional layer of defense against predatory insects and the invasion of plant pathogens. Several beneficial bacteria have been documented to exhibit antagonistic activity against microorganisms harmful to plants, through either competition or the synthesis of antimicrobial biomolecules. To confirm their antagonistic properties, conducting experiments on bacteria under laboratory, greenhouse, and field conditions is crucial. NPs can improve the selected bacteria for biocontrol [37]. However, it is hard to estimate whether NPs can promote plant pathogens. Therefore, substantial research on bacteria, nanoparticles, plant pathogens, and plants is required to ensure the effectiveness and safety of nanobiofertilizers. There is a shortage of studies on molecular interactions between plant-growth-promoting bacteria, NPs, and plants.

The molecular activity of plants induced by nanomaterials linked with plant-growth-promoting bacteria (PGPB) is still not completely understood now. Previous research [17, 38, 39] on nanoparticles and plants has demonstrated that smaller NPs can move across the symplast through plasmodesmata, but larger NPs have a greater propensity to aggregate in the intercellular space. However, these trials did not include bacteria, and the results may be different when PGPB is introduced to the system. Rhizophagy is a process that plants use to get nutrients by ingesting microorganisms and decomposing them inside the plant's roots [40]. According to study [40], plant roots are responsible for producing ROS, which destroys the cellular proteins, cell wall, cell membranes, and various cellular components of beneficial microbes present in the periplasmic spaces of the plant. It allows it to harvest nitrogen from the bacteria. Research provides evidence that plants engage in the process of microbivory to get nutrients [22, 41]. When all the plant's nutrients have been used, the microorganisms that have survived will exit the plant via its root hairs to repopulate the rhizosphere and begin the process again. This suggests that few NPs associated with bacterial cell walls can linger in the plant tissues even after removing the bacteria [38].

## 4. Bioinoculants and Nanoparticles: Strengthening Plant Resistance against Biotic and Abiotic Stresses

Bioinoculants and nanoparticles play a significant role in enhancing plant resistance against biotic and abiotic stresses [19, 46]. Biotic stresses refer to the harmful effects caused by living organisms such as pathogens, pests, and weeds, while abiotic stresses include environmental factors like drought, salinity, extreme temperatures, and heavy metal contamination. Both types of stresses can severely impact plant growth, development, and overall productivity [47]. However, the application of bioinoculants and nanoparticles has shown promising results in improving plant resilience and reducing the detrimental effects of these stressors.

Bioinoculants establish nonsymbiotic or symbiotic relationships with plants, promoting growth, nutrient uptake, and defense mechanisms [27]. One of the key ways bioinoculants enhance plant resistance to biotic stress is through induced systemic resistance (ISR). ISR involves the activation of defense responses in plants by beneficial microorganisms, providing protection against pathogens [48]. The microorganisms can produce antimicrobial compounds, stimulate plant hormone production, and compete with pathogens for nutrients and space, thereby reducing disease incidence and severity [4]. In addition to biotic stress management, bioinoculants also contribute to plant resistance against abiotic stresses. Certain microbial strains possess traits that enable them to thrive in harsh environments and improve plant tolerance to abiotic stressors. For example, some bacteria and fungi have the ability to solubilize nutrients, enhance nutrient availability, and promote plant growth under nutrient-deficient conditions [27, 45, 49]. They can also produce stress-responsive proteins and enzymes that help plants withstand drought, salinity, and temperature extremes. Furthermore, certain microbial strains can facilitate the uptake and translocation of essential minerals, enhancing plant nutrition and overall stress tolerance [50].

Nanoparticles (NPs), when applied to plants, can interact with plant tissues, organelles, and cellular components, triggering specific responses that improve stress tolerance. NPs can act as carriers for delivering bioactive compounds, such as antioxidants and hormones, directly to the plant cells, thereby mitigating the damaging effects of stress [47, 51]. Moreover, NPs can modulate plant hormone signaling pathways, promoting growth and development even under stressful conditions. Nanoparticles also have the ability to scavenge reactive oxygen species (ROS), which are generated as byproducts of stress and can cause cellular damage [27]. By reducing ROS levels, NPs help protect plants from oxidative stress and maintain cellular homeostasis. Additionally, NPs can enhance the efficiency of photosynthesis by improving light absorption and utilization, thereby enhancing plant growth and productivity.

### 4.1. Salinity and Drought Abiotic Stress

Predictions indicate a rise in the frequency and severity of droughts, which could significantly impact global crop yields. This form of nonliving stress has a profound influence on soil organisms and plants, leading to osmotic stress and a decrease in nutrient availability for plant roots [46]. Additionally, soil salinity is a prevalent nonliving stressor that greatly affects crop growth and yield, resulting in substantial economic losses across 1125 million hectares, with salinization and sodification causing the loss of 1.5 million hectares of cultivable land annually [19]. Salt stress compromises the integrity of the plasma membrane, diminishes photosynthetic efficiency, and reduces the opening and accessibility of stomata and antioxidant enzymes, leading to an overproduction of reactive oxygen species (ROS), which in turn affects proteins, DNA, and lipids [46, 51, 52]. Ionic stress arises from an excessive accumulation of sodium (Na^+^) and chloride (Cl^−^) ions in plants, disrupting the distribution, absorption, and availability of macro- and microelements, as well as the integrity and selectivity of cellular membranes [27]. Nanoparticles (NPs) have been shown to have beneficial effects on plants under drought and salinity stress by altering phytohormone levels, gene expression, and secondary metabolite production; enhancing nutrient concentration and availability; reducing plasma membrane damage and chlorophyll degradation; improving K+ uptake and K+/Na+ ratio; and boosting antioxidant enzyme activity [7, 25, 27, 46, 47]. Thus, NPs help alleviate drought-induced ROS by aggregating osmolytes, leading to improved osmotic adaptation and crop water balance. Moreover, NPs enhance photosynthetic activity, upregulate aquaporins, alter intracellular water metabolism, accumulate compatible solutes, maintain intracellular ion balance, increase stomatal density, and decrease water loss from leaves through stomatal closure due to increased ABA accumulation [50, 53, 54].

### 4.2. Temperature Elevation Abiotic Stress

The escalation of environmental temperature is a result of the emission of greenhouse gases into the atmosphere. This has led to a consistent rise in the average global temperature, which is projected to increase by 2°C by the year 2100 [48]. This increase is expected to cause significant losses in agricultural production across the globe. Greenhouse gases have multiple impacts on crop growth and development. A high concentration of CO_2_ typically boosts photosynthesis, leading to enhanced plant growth and productivity. However, the rise in temperature negates this benefit by increasing evapotranspiration and the rate of crop respiration. This also encourages the proliferation of pests and weeds, shortens the crop growth period, and negatively impacts the soil's microbial population and enzymatic activities [27].

To cope with heat stress, which can lead to oxidative stress due to an excess production of reactive oxygen species (ROS), plants undergo morphological and biochemical changes. These changes can severely impact their growth, development, and yield. In response to such abiotic stress, plants initiate signaling processes to produce osmolytes, which help maintain cell turgidity through osmotic adjustment, and other secondary metabolites to enhance the antioxidant system [45, 55–57]. High temperatures can harm photosynthetic functions by damaging the oxygen-evolving complex, PSII cofactors, carbon assimilation, and ATP production. In contrast, low-temperature stress can cause freezing injury and reduce fluidity in cell membranes. The positive influence of nanoparticles (NPs) on plants under temperature stress has been shown. They enhance photophosphorylation, oxygen evolution, and water-splitting CP43 protein; improve nitrogen metabolism and photosynthetic capacity; increase antioxidant enzyme activity; decrease lipid peroxidation; and restore the ultrastructural distortions of chloroplasts and the nucleus [26, 53, 58, 59].

### 4.3. Heavy Metal Contamination Abiotic Stress

The presence of harmful heavy metals (HMs) and metalloids, such as arsenic (As), lead (Pb), cadmium (Cd), copper (Cu), chromium (Cr), manganese (Mn), mercury (Hg), nickel (Ni), selenium (Se), antimony (Sb), and zinc (Zn), in soil is a significant environmental problem with adverse effects on all living organisms [50]. The primary contributors to the accumulation of these substances are human activities, including industrial and municipal discharges, mining, smelting, improper disposal of hazardous solid waste, and extensive use of agrochemicals. These HMs persist in the soil for extended periods due to their nonbiodegradable nature. They can be absorbed by crops, eventually reaching human consumption, and leading to various health issues such as cancer (resulting from DNA damage), cardiovascular problems, dermal diseases, gastrointestinal disorders, respiratory damage, brain damage, kidney disorders, degenerative bone diseases, liver damage, depression, mental retardation, and respiratory system disorders [60].

The mobility and availability of HMs in the soil are controlled by biogeochemical processes such as mineralization, precipitation, adsorption, and protonation, as well as by factors like soil type and the rhizosphere—a region around the roots containing a diverse root microbiome that contributes to soil fertility but is highly affected by these compounds [61, 62]. To mitigate the negative effects of this abiotic stress, several mechanisms have been proposed. One aspect worth highlighting is the use of nanoparticles (NPs) in soil remediation, which can immobilize metal ions through absorption, oxidation, or chemical reduction processes [56]. In plants, NPs can influence the formation of apoplastic barriers that regulate the movement of water, ions, and oxygen, thereby reducing the accumulation of HMs in the roots. Specific NPs can also regulate the expression of metal transport genes, enhancing the plant's extracellular barriers to intercept HMs [63]. Organic acids present in the cell walls of roots and leaves can chelate with HMs, reducing their damaging effects on plants under HM stress. Additionally, NPs can activate the oxidation defense system, which helps alleviate HM stress [50, 60].

It is worth noting that the use of bioinoculants and nanoparticles for plant stress management is an area of active research, and their efficacy may vary depending on the plant species, stress conditions, and application methods. Nevertheless, the potential of these technologies in enhancing plant resistance to biotic and abiotic stresses holds great promise for sustainable agriculture, enabling improved crop yields, reduced pesticide usage, and enhanced ecosystem resilience. Continued research and development in this field are crucial to fully harness the benefits of bioinoculants and nanoparticles in addressing the challenges posed by biotic and abiotic stresses in agriculture.

## 5. Production of Nanoparticles on an Industrial Level

**Figure 2:**
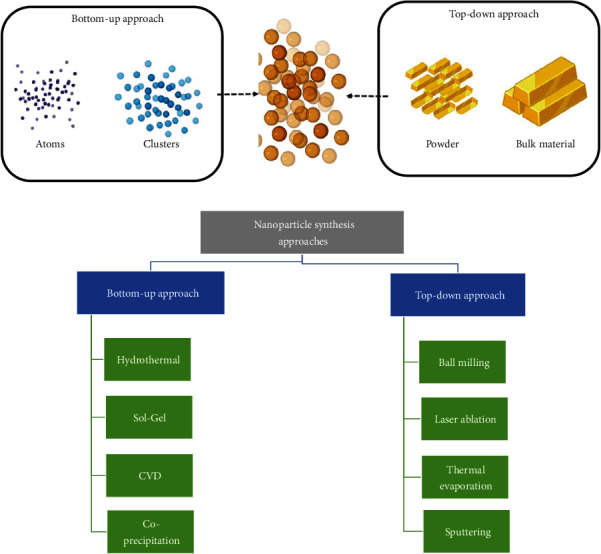
Bottom-up and top-down nanoparticle synthesis approaches used for nanoparticle synthesis.

Bottom-up and top-down methods are the two primary techniques that may be utilized to produce industrial nanoparticles [64]. Methods that start from the bottom up require an intricate and highly organized arrangement of atoms, which leads to the formation of different structures, i.e., nanoparticles and clusters (Figure 2). Top-down techniques, on the other hand, suggest that the nanostructure is produced by removing atoms or crystallographic planes from the initial material (Figure 2). Both have their merits and some drawbacks [21]. The ball-milling technique is the top-down method used most frequently to produce nanoparticles. This method has the advantage of producing large numbers of nanoparticles from low-cost starting materials, although it also has the disadvantage of producing nanoparticles with irregular shapes and wide particle size dispersion. Ball milling is a procedure that may be used to treat a variety of oxides, including various phosphates.

Some top-down methods, like electron beam trigger etching, typically produce a few milligrams or little usable nanomaterial, limiting their relevance due to less amount and high cost. Bottom-up techniques are simple and usually utilize water as a solvent, which benefits the environment. Water-soluble salts may be reduced by less expensive molecules, such as citrates or alcohols, to produce metallic nanoparticles relatively easily on a large scale. Examples of these nanoparticles include silver and gold [13]. The production of metallic nanoparticles may now be done in a less harmful way to the environment thanks to natural extracts. When used in situations where they may serve as both a solvent and a reactant, nonaqueous solvents have made possible a novel approach to the production of nanoparticles. The hydrolysis of certain alkoxides can be used on an industrial scale to produce silica and other oxides [36, 65]; however, rather than utilizing one big reactor, it is more common to use a number of smaller reactors.

## 6. Major Nanoparticles Used in Agriculture

### 6.1. Zinc Oxide Nanoparticles

Zinc oxide (ZnO) is a form of *n*-type semiconductor [66]. The qualities of zinc oxide nanoparticles include strong chemical stability, nontoxicity, biocompatibility, and photothermal stability. Production of zinc oxide nanoparticles is also very inexpensive. Zinc oxide has the potential to be an effective photocatalyst due to its excellent ability to absorb UV energy [67]. Because of its bandgap, which is 3.37 eV, this chemical is frequently used as a substitute for titanium dioxide (TiO_2_). Because of their biocompatibility, biodegradability, and antibacterial qualities, zinc oxide nanoparticles have several applications in the pharmaceutical and food-packaging industries [67, 68]. In addition, ZnO possesses piezoelectric capabilities, which allow it to generate electrical tension when subjected to mechanical pressure. These qualities make ZnO an attractive candidate for use in sensors. The structure of these nanoparticles determines whether they are 1-, 2-, or 3-dimensional; i.e., 1-dimensional nanoparticles include nanowires, nanotubes, nanoneedles, and nanorods. Three-dimensional nanoparticles include examples such as graphene and carbon nanotubes [66]. A nanosheet is an example of a 2-dimensional ZnO particle, while a flower is an example of a ZnO particle that is 3-dimensional. Synthesis of ZnO NPs can be accomplished by either chemical or physical means. Two widely utilized physical techniques are physical vapor deposition (PVD) and laser ablation. PVD entails the transfer of material in a vapor state onto a substrate, while laser ablation involves the removal of atoms from a material using a high-powered laser beam [69]. The chemical processes of precipitation, sol-gel, and hydrothermal synthesis are the ones that are used most frequently. The precipitation synthesis method involves reducing the zinc salt with the chemical solvent that manages NP size, subsequently heating the produced powder [49]. Zinc chloride and zinc acetate are the two forms of zinc utilized in this process. Ammonium carbonate is the reducing agent that is applied.

In addition to making it feasible to perform surface modifications, the sol-gel process makes it possible to create ZnO nanoparticles in a manner that is both repeatable and inexpensive [49]. The composition of the reaction mixture may be altered to produce materials with a high degree of crystallinity, which enables the hydrothermal approach to be employed for the synthesis of nanoparticles of a wide range of sizes and forms. Agriculture has benefited from using ZnO NPs, which have been shown to boost crop growth and increase yields [18, 70]. For instance, the presence of ZnO NPs in peanut (*Arachis hypogaea*) plants at a concentration of 1000 ppm boosted root and stem development and promoted seed germination and seedling vigor [71]. Various effects might occur due to these NPs interacting with PGPB. In 24 hours, the bacteria *Pseudomonas chlororaphis* with ZnO NPs at a concentration of 500 mg/L produced a greater quantity of siderophores than the controls. NPs, which bind to siderophores, might be responsible for releasing Zn^2+^ ions, which would explain this phenomenon [72].

On the other hand, ZnO was proven effective in preventing IAA development after 48 hours. However, it has been demonstrated that the release of cations cannot be responsible for this decrease because it does not affect the synthesis of IAA when Zn^2+^ is present in the solution [72]. In addition, the ZnS PGPB species *B*. *amyloliquefaciens*, *P. fluorescens*, and *P. aeruginosa* had their IAA synthesis suppressed by zinc oxide nanoparticles. Researchers [61] employed NP concentrations ranging from 100 g/mL to 400 g/mL and reported that the higher NP concentrations decreased productivity. In addition, the NPs could prevent the phosphate liquefication activity by *B*. *amyloliquefaciens*, *P. fluorescens*, and *P. aeruginosa*, which, once more, was shown to be dependent on the zinc oxide content. Regardless, the presence of ZnO nanoparticles caused all the bacteria to create more siderophores than they did under the control circumstances, and a rise in the concentration of the NPs caused an increase in the synthesis of this molecule. In another research [73], it was observed that ZnO nanoparticles with PGPR increase the soybean plant's grain weight, the number of nodules, and the plant height. The dry weight of nodules produced by each plant, the number of pods produced by each plant, and the number of grains produced by each plant rose when the concentration of zinc oxide was raised. Similarly, ref. [74] observed that ZnSO_4_ NPs, when combined with the *Pseudomonas* spp. PGPB, boosted the levels of zinc, potassium, phosphorus, and nitrogen in rice plants. This led to an increase in grain production as well as an increase in seed nutrient content.

### 6.2. Silicon Dioxide Nanoparticles

Silicon, the next most prevalent element in nature after O_2_, is ubiquitous in soil. Plants have an innate requirement for silica because it supports them in responding to abiotic and biotic stresses. These interactions increase plant water usage, photosynthesis potential, structural properties, and rigidity, which protects leaves from falling over and shields them from diseases [62, 75]. Plants also require silica because it helps them react to biotic and abiotic stresses. Plants acquire dissolved silica from the soil water and deliver it to various cells/tissues via the vascular systems, which are used to build silica bodies. Nanoparticles composed of silicon oxide, often known as silica, are one of the most utilized types of nanoparticles, SiO_2_ nanoparticles in particular [76]. They might be produced from their natural surroundings, or they can be synthesized in laboratories. Artificial nanoparticles are amorphous, while extracted nanoparticles are mineral silica with a crystalline phase, like quartz. The merit of synthesizing such NPs is the purity of the finished product, while extracted NPs include metal impurities.

Significant quantities of silica can be generated, and the morphology, dimensions, extensive surface area, chemical inertness, and biocompatibility of silica nanoparticles (NPs) can be controlled. As a result, silica NPs have found widespread application in various fields [66]. One of the most important applications for these materials is in controlled-release systems, which enable the loading of a wide variety of substances, including pharmaceuticals, DNA, RNA, proteins, fertilizer components, and pesticide constituents. In addition to enabling the regulated release of chemicals, the efficiency, specificity, biocompatibility, and bioactivity of molecules can be improved using silica nanoparticles (NPs). Manufacturing mesoporous silica NPs can help regulate discharge since the pores prevent molecules from leaving [7]. It has also been reported that the usage of silica nanoparticles in agriculture can boost the survival of maize seeds when they are included in preparations of smart pesticides and can enhance bioremediation [36]. The most prevalent techniques for synthesizing SiO_2_ nanoparticles are reverse microemulsion, FAME synthesis, and the sol-gel technique. A surfactant that has been dissolved in an organic solvent can be used in a process called reverse microemulsion synthesis to create spherical micelles [2]. When the polar heads meet water, they begin to arrange themselves into what is known as reverse micelles. These micelles are microcavities that hold water. After the addition of the silicon precursor, the nanoparticles are then able to penetrate these microwells. The high expenses involved and the difficulty in removing the surfactant from the final material are two drawbacks of this approach. Flame synthesis, often called chemical vapor condensation, is an approach to chemical synthesis predicated on the flame breakdown of metal-organic precursors. This approach has several drawbacks, including the difficulty of managing the NP size and shape. The sol-gel method includes hydrolyzing and then condensing a silicon precursor called alkoxide. This procedure requires an acidic or basic catalyst. The Stober technique is the one that is utilized for sol-gel synthesis the most [77]. Tetraethyl orthosilicate (TEOS, Si(OC_2_H_5_)_4_) is the utilized precursor, and it is incorporated into a solution that already consists of water and ethanol [78]. During hydrolysis, an ammonium hydroxide base catalyzes the water nucleophilic action on the alkoxide. This results in the formation of silanol groups, which have the formula Si(OH)_4_. These groups condense into Si–O–Si chains, which culminate in forming the three-dimensional structure of silica.

The creation of scattered NPs with a spherical form and the capacity to adjust their size are two of the most significant benefits of using the Stober approach. The concentration of the chemicals and the temperature play a role in determining the NP sizes produced by the sol-gel synthesis technique. Their final sizes are determined by the molar ratio of TEOS to NH_3_; the greater the ratio, the more condensed the molecule, and the smaller the diameter. Ref. [79] used chemometrics to conduct research in which they investigated the effect that every individual chemical had on the produced size of the NPs. It was observed that during the experiment in which the total amount of ammonia was raised, the NPs that were ultimately harvested likewise grew. The production of mesoporous silica begins with the addition of a surfactant to the reaction mixture, followed by the heating of the mixture and the subsequent addition of TEOS. CTAB (cetrimonium bromide) is the surfactant that is utilized the most frequently, and the temperatures are near 80 degrees Celsius [80]. An experiment that was carried out using maize (*Zea mays*) seeds indicated that 50 nm silica NPs improved the population and survivability of PGPB in the soil. The NPs did not demonstrate any harmful effects, allowing the bacteria to thrive at their ideal pH. In addition, they raised the total amount of nitrogen, phosphate, and potassium (NPK), which caused the maize seeds to all germinate successfully [53]. The nanosilica effect on PGPB levels in maize was also investigated by [55]. According to the findings, the nanoparticles increased the bacterial population and the soil's total biomass and nutritional content. In each investigation, silica nanoparticles were demonstrated to be superior to other types of silicon sources. It was shown that tomato plants (*Lycopersicum esculentum*) benefited from nanosilica by reducing mean germination time, enhancing seed germination rate, increasing seedling fresh weight, and reducing seedling dry weight. In each of these trials, the germination rates were higher, which points to the fact that silica NPs have the potential to boost agricultural yields.

Because of their encapsulating ability, nanoparticles have yet another potential application in agriculture. *Pseudomonas fluorescens* and *Bacillus subtilis* were encased in alginate silica NPs and carbon nanotubes in a study conducted by [81]. Pistachio UCB-1 has its root length, and this procedure improved its ability to be micropropagated. Inoculation of explants with bacteria inside of capsules increased plant biomass and bud length compared to the controls. Ref. [82] examined the silica NPs extracted from *Equisetum telmateia* with phosphate-solubilizing rhizobacterium *Mesorhizobium* spp. and *Pseudomonas stutzeri* on the growth of land cress (*Barbarea verna*). The application of the NPs at concentrations of 0.05 and 0.07 ppm improved the growth of the bacteria. Using NPs in conjunction with both kinds of bacteria resulted in the highest recorded dry weights of the shoots and roots and an increase in the amount of nitrogen and phosphate contained in the soil. This led to an improvement in the growth of the plants. Silica nanoparticles with a size range of 5–20 nm have also been demonstrated to have a beneficial effect on *B. subtilis*, leading to an increase of 85% in cytokinin production [76]. The hydrating characteristics of silica NP surfaces may be used to explain the interaction between nanosilica and bacteria. These features make it easier for bacteria to be attracted to nanosilica, resulting in improved bacterial acid resistance. In addition, studies [69, 83, 84] have shown that the presence of silicon dioxide particles in the medium leads to an upsurge in the negative surface charge possessed by some gram-negative bacteria. The charge density elevation is induced by a change in porin conformation caused by particle adsorption onto cell surfaces. According to several studies [58, 76], nanoparticles of SiO_2_ can improve bacteria's capacity to stimulate plants' development. There is a possibility that the increased oxygen mass transfer and ion exchange activities in the medium are responsible for the stimulating effect that mineral NPs have on the development of bacteria. In addition, the attachment of NP to the bacteria's surface may change the cells' shape and size, leading to increased proliferation.

### 6.3. Titanium Dioxide Nanoparticles

One of the most popular semiconductors with photocatalytic activity is titanium dioxide (TiO_2_), an oxide generated from a transition metal [85]. It is also used in various household items, including food-packaging materials, cosmetics, ceramics, plastics, paper, and paint [63]. Its chemical stability, safe handling, and biocompatibility are among the factors for its extensive usage. There are three distinct crystalline forms of titanium dioxide [86], and they are called anatase, rutile, and brookite. Anatase and rutile both have a tetragonal architecture. Anatase is favored over rutile regarding the photocatalytic performance of TiO_2_ since anatase has better transitional states (Fermi level) and adsorbs less oxygen than rutile. However, this material can only be triggered by ultraviolet rays since its energy bandgap is near 3.0 eV and fluctuates for every phase. TiO_2_ nanoparticles have a higher photocatalytic capability than bulk TiO_2_ because they have a larger surface area. Different production processes may produce titania nanoparticles, including sol-gel, hydrothermal, oxidation, and deposition processes. The primary prevalent strategies for altering the size and form of nanoparticles are hydrothermal and sol-gel approaches.

In most cases, the synthesis is conducted in an autoclave that is strong enough to endure the extreme physical and chemical circumstances applied. Advantages include size, purity, and minimal agglomeration. Sol-gel synthesis is a wet chemical method for producing high-purity nanoparticles at reduced temperatures and controlling doping stoichiometry. In this technique, a titanium precursor is dissolved in a solution, producing an inorganic solid composed of nanoparticles [82]. The precursor can be hydrolysed or solubilized, producing a solution (sol) composed of microparticles distributed in a liquid. Condensation then causes the precursor to transform into a gel. Metal oxides and metal chlorides are both examples of common precursors. According to Park et al.'s [56] findings, *Pseudomonas* spp. exhibited higher adherence and bioluminescence levels on surfaces containing nanostructured titania compared to normal titania. Therefore, it is reasonable to postulate that nanotitania could be capable of directing bacteria to a particular location. It has been established that plant-growth-promoting rhizobacteria (PGPR) may more easily adhere to plant roots with the assistance of TiO_2_ nanoparticles produced using the sol-gel process. When bacteria are cultivated in the vicinity of nanoparticles, the resulting biofilm is more persistent and has a greater thickness than when cultivated within a self-produced biofilm. Titanium dioxide (TiO_2_) has many applications, including in agriculture [17, 87]. In agriculture, TiO_2_ is used as a photocatalyst to promote plant growth and improve soil quality. TiO_2_ can also be used to remediate contaminated soil, making it safe for growing crops. Use of TiO_2_ nanoparticles in agriculture is based on photocatalysis principles, which involves converting light energy into chemical energy. It has been observed that TiO_2_ can support PGPR in adhering to plant roots and establishing persistent and stronger layers than in a biofilm. This increased bacterial colonization leads to improved plant growth, biomass, and crop yields [46]. For example, the biomass of wheat seedlings increased when repeated inoculations of PGPR were administered with TiO_2_ nanoparticles in peat soil under unfavorable conditions induced by drought, salt, and fungal infections. TiO_2_ also helps protect plants against fungal pathogens [88]. For example, PGPB *Bacillus amyloliquefaciens* attaches to oilseed rape (*Brassica napus*) roots in the presence of TiO_2_ and protects the plants against the fungal pathogen *Alternaria brassicae* [19].

TiO_2_, combined with rhizobacterium, has also been studied for its potential to enhance phytoremediation in cadmium- (Cd-) contaminated soils. When TiO_2_ nanoparticles were applied with the rhizobacterium *Pseudomonas fluorescens*, white clover (*Trifolium repens*) seedlings showed improved plant growth and increased plant biomass up to 500 mg/kg [25]. Furthermore, both the root and shoot Cd absorption and accumulation capacities improved. It has been shown that broad bean plants grown in saline soil can benefit from TiO_2_ nanoparticles by enhancing either the enzymatic activity of the plants or the solubility of sugars and amino acids in the soil. These findings demonstrate that TiO_2_ nanoparticles can help grow crops in contaminated soils. In addition to improving plant growth and soil quality, TiO_2_ is also used to remediate contaminated soil. TiO_2_ is a photocatalyst that can break down organic pollutants in soil into nontoxic byproducts [86]. The photocatalytic degradation process involves the absorption of light by the TiO_2_, which excites electrons and causes them to transfer to the conduction band. This creates electron-hole pairs, generating reactive species, such as hydroxyl radicals, that can break down pollutants. TiO_2_ effectively breaks down various pollutants, including herbicides, pesticides, dyes, and phenols. TiO_2_ is a versatile material with many applications in agriculture. Its use as a photocatalyst has led to improved plant growth and increased crop yields, and it can also be used to remediate contaminated soil, making it safe for growing crops. TiO_2_ nanoparticles have a greater photocatalytic capacity than bulk TiO_2_, which makes them more reactive and effective in promoting plant growth, improving soil quality, and remedying contaminated soil. TiO_2_ in agriculture is an example of how materials science and technology can be applied to solve important agricultural challenges and improve food security.

### 6.4. Other Materials as Nanoparticles

Because of their proximity to these systems, natural mineral nanoparticles are abundant in soils and perform a vital function in microbes' metabolic and physiological activities. As a result, research into nanomaterials' effects on PGPB has been conducted to enhance the efficiency with which bacteria function in plants [93]. The researchers in [52, 86, 94] showed that vermiculite and saponite nanoparticles (NPs) stimulated bacterial proliferation at 1.5 and 2.5 g/L values, respectively. They also observed that vermiculite NPs boosted dehydrogenase function in *Azotobacter vinelandii* and peroxidase function in *B. subtilis*. Wheat and barley plants benefited from using a mixture that included bacteria and nanomaterials. Not only did it increase grain production and protein content, but it also decreased lesions caused by illnesses.

Furthermore, ref. [94] reported that the vermiculite NPs enhanced abscisic acid (ABA) yield in *B. subtilis* and *A. vinelandii*, in addition to IAA synthesis in *B. subtilis*. The ABA phytohormone regulates a plant's resistance to both abiotic and biotic stresses, whereas the phytonutrient known as IAA is necessary for plant growth and development. The combination of mineral nanoparticles (NPs) with plant-growth-promoting bacteria (PGPB) holds great promise for enhancing plant growth, resilience against adverse conditions, and protection against plant diseases. This approach is considered safer and has significant potential for future applications, as both mineral NPs and silica are naturally occurring substances [95, 96]. Therefore, studying how various bacterial species interact with NPs of differing shapes, sizes, and amounts in their natural settings is essential. At a concentration of 200 mg/L, the nanoparticles of CuO caused an increase in the amount of IAA that was synthesized by the plant-growth-promoting bacteria *Pseudomonas chlororaphis*, which the release of ions may explain [32, 72]. Depending on the dosage administered, these NPs can harm beneficial plants and microorganisms. As a result, the significance of this finding lies in the nanomaterial's dose affecting the bacteria. Adding copper oxide nanoparticles (CuO NPs) to the rhizosphere of wheat (*Triticum aestivum*) led to enhanced microbial population health, heightened nitrogen fixation rates, and reduced denitrification rates. These combined effects synergistically promoted the growth and development of the plant [97]. There was shown to be a connection between elevated levels of nitrate in the rhizosphere and elevated levels of gene expression associated with nitrogen fixation. Based on these findings, it seems likely that CuO NPs might have a role in gene expression connected to N_2_ oxidation.

Gold nanoparticles (AuNPs) are desired due to their inert chemical nature, tolerance to surface oxidation, and nontoxicity in natural environments. Shukla et al. [59] recommended the inclusion of AuNPs in nanobiofertilizer formulations due to their growth-promoting effects on advantageous *P. fluorescens* and *B. subtilis*. Manufacturing a good nanobiofertilizer may also be accomplished by combining more than one type of bacteria with several nanomaterials. In spring wheat plants, Mardalipour et al. [98] found that there were benefits linked with several NPs that were connected with two PGPBs. The nanobiofertilizer, named Biozar®, is formulated with *Azotobacter*, *Pseudomonas*, iron, zinc, and manganese nanoparticles. It was found to improve agronomic qualities and boost crop growth and production. AgNPs are effective at preventing and treating a variety of plant ailments. These particles stimulate growth and prevent ROS-induced senescence in stressed plants. Ref. [54] examined the impact of AgNPs on Brassica (*Brassica juncea*), cowpea (*Vigna sinensis*), and wheat (*Triticum aestivum*) rhizobacterial biodiversity and development. Cowpea responded favorably regarding development parameters and root nodulation, while Brassica did so regarding shoot parameters. Wheat, on the other hand, showed only a negative effect. In cowpea and wheat, the concentration of 75 ppm decreased the population of N_2_ fixers and siderophore makers, but the concentration of 50 ppm had a beneficial effect on P solubilizers. This was in relation to bacterial diversity. In the case of Brassica, there was no discernible difference in the level of biodiversity produced by either concentration. According to research by [99], AgNPs exhibited bactericidal effect towards N_2_-fixing, biofilm-forming, and phosphate-solubilizing bacteria at concentrations as low as 2–22 g/mL when the compounds were tested.

On the other hand, there was a rise in the number of bacteria belonging to the species *Stenotrophomonas* spp. and *Pseudomonas* spp., both known for their ability to stimulate the development of plants. Ref. [60] performed a combined experiment to determine the impact of PGPR (*Bacillus cereus* and *Pseudomonas* spp.) and AgNPs on the growth of maize. The NPs attached to the bacteria surface could help to increase the root area as well as the root length. Rhizobacterial bioremediation abilities for lead, cadmium, and nickel increased due to nanosilver particles. It has also been found that AgNPs containing *Pseudomonas putida* are beneficial to cucumber (*Cucumis sativus* L.) plants [100]. These nanoparticles improved the plant's resistance to disease and stress by enhancing the activity of the enzyme's catalase (CAT), superoxide dismutase (SOD), and phenylalanine ammonia-lyase (PAL), as well as increasing the flavonoid content of cucumber leaf tissue. It is important to point out that although these findings demonstrate that AgNPs can stimulate plant development, they should also be considered due to their potentially harmful effects on specific bacterial populations. Several studies have revealed that AgNPs exhibit bactericidal action, and this activity negatively influences the variety of soil microorganisms. However, these NPs' toxicity appears to depend on the bacterial species and procedures used in their production.

## 7. Nanoparticle Implications on Plant-Growth-Promoting Bacteria

**Figure 3:**
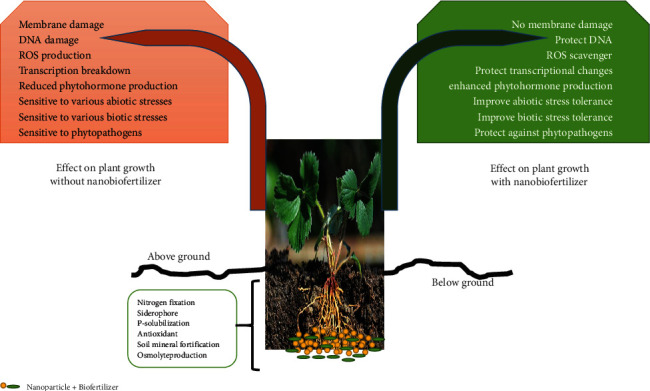
The impact of nanobiofertilizers have on plants while they are under stress.

Although nanoparticles can potentially increase the population of PGPB in soil, there is still a chance that they will impact soil microbes. It appears that the form of the NPs is one of the elements that influence their biological properties. For instance, in [101], it was found that triangular AgNPs displayed the strongest potent bactericidal activity on *Escherichia coli*, whereas [72] reported that round silver nanoparticles did not affect the *Pseudomonas chlororaphis* membranes integrity. Thus, it has been hypothesized that differently shaped AgNPs with equivalent surface areas could exert different biological effects. This is true even if the surface areas are the same. In a separate piece of research, ref. [32] found that the presence of CuO NPs increased the amount of bacterial IAA production, but the presence of ZnO NPs decreased the phytohormone amount in the same bacteria. Few researchers hypothesized that NPs of various forms were essential to forming secondary metabolites by bacteria [43, 48, 61].  Figure 3 summarizes the influence of nanobiofertilizers on plants during periods of stress.

It would appear that the kind of metal and the concentration of the NPs both affect the toxicity that bacteria experience. Ref. [99] found that TiO_2_ nanoparticles were more inhibitive to gram-positive bacteria and disturbed the integrity of bacterial cell membranes with enhanced toxicity. The findings of [57] demonstrated that soil bacteria were more sensitive to the toxicity of Cu and Zn in their more soluble forms than in their metal oxide or nanoforms. Consequently, these NPs can potentially be more hazardous under the right conditions than the bulk material. However, this is only the case if this form's metal dissolution is more significant. Nanoparticles that have a positive electrical charge are highly detrimental to most biological systems, NP toxicity is linked to ionic dissolution, and asymmetrical and rod-shaped nanoparticles are especially hazardous, even though they are absorbed less readily. These findings make up the overall conclusion about the toxicity of nanomaterials. In terms of their phytotoxicity, NPs can have good or detrimental effects on plants [30, 102]. The type of plant, its species, and the NP's physicochemical properties (like concentration, size, type, and exposure time) play a role in the hazardous effects. The release of nanomaterials into the environment can potentially be detrimental to living creatures on many levels. The many factors pose the most significant barrier towards the application of NPs in agriculture. These factors include the material composition of the nanomaterials, as well as their size, form, amount, and interactions. It is essential to note that the combination of NPs and PGPB has resulted in agricultural advancements. Therefore, there is an immediate need for in-depth research into the fatal and sublethal dosages that should be conducted on a case-by-case basis [103].

## 8. Consequences of Metal Uptake in the Plant and Its Effects on Humans

Metals play a crucial role in the growth and development of plants, as they are essential micronutrients required for various biochemical processes. However, excessive metal uptake by plants can have significant consequences both for the plant itself and for human health when these plants are consumed. In this detailed overview, we will explore the consequences of metal uptake in plants and its potential effects on humans [46, 85, 104, 105].

### 8.1. Consequences of Metal Uptake in Plants

#### 8.1.1. Plant Health

- *Toxicity*: excess uptake of certain metals, such as cadmium (Cd), lead (Pb), and mercury (Hg), can be toxic to plants. These metals interfere with essential physiological processes, including photosynthesis, respiration, and enzyme activity, leading to reduced growth, wilting, and even death [104]
- *Nutrient imbalance*: high metal concentrations can disrupt the balance of essential nutrients in plants [104, 106]. For example, excessive iron (Fe) uptake can inhibit the absorption of other essential metals like manganese (Mn) and zinc (Zn) [107]

#### 8.1.2. Environmental Impact

- *Bioaccumulation*: plants that accumulate high levels of heavy metals can become a source of environmental contamination. These metals can leach into the soil, affecting nearby ecosystems and water bodies, potentially harming aquatic organisms and wildlife [107, 108]
- *Soil degradation*: metal-contaminated plants can contribute to soil degradation, making it unsuitable for agriculture or other land uses. This can have long-lasting ecological and economic consequences [108]

#### 8.1.3. Food Chain Contamination

- *Bioavailability*: plants serve as a critical link in the food chain. When metal-contaminated plants are consumed by herbivores, the metals can biomagnify as they move up the food chain, ultimately affecting predators and humans [103, 105, 109]
- *Human Exposure*: crop plants contaminated with heavy metals can lead to the direct consumption of these metals by humans, posing a significant health risk [110, 111]

### 8.2. Effects on Human Health

#### 8.2.1. Direct Ingestion

- *Acute poisoning*: ingesting plants with high levels of toxic metals can lead to acute poisoning [111]. For example, consuming vegetables contaminated with high levels of lead or cadmium can cause immediate symptoms such as nausea, vomiting, and abdominal pain [111, 112]

#### 8.2.2. Chronic Exposure

- *Long-term health effects*: chronic exposure to low levels of metals through the consumption of contaminated plants can lead to various health problems over time [113, 114]. The following are examples:
- *Cadmium*: long-term exposure to cadmium has been associated with kidney damage, osteoporosis, and an increased risk of certain cancers
- *Lead*: chronic exposure to lead can result in developmental and cognitive impairments, especially in children, as well as cardiovascular and neurological issues in adults
- *Mercury*: long-term exposure to mercury, especially the highly toxic methylmercury found in some fish, can damage the nervous system and harm fetal development during pregnancy

#### 8.2.3. Cumulative Effects

Metals can accumulate in the body over time, and even low-level exposure to multiple metals simultaneously can have synergistic effects [111], increasing health risks [115].

#### 8.2.4. Children and Vulnerable Populations

Children, pregnant women [112, 114], and individuals with compromised immune systems or underlying health conditions are particularly vulnerable to the adverse effects of metal exposure [116].

The consequences of metal uptake in plants can have far-reaching effects on both plant health and human well-being [117]. It is essential to monitor and manage metal contamination in agricultural practices to minimize the risk of human exposure to toxic metals through the food chain. Sustainable agricultural practices, soil remediation techniques, and stringent regulations are some of the strategies used to mitigate these risks and ensure food safety [114, 117].

## 9. Limitations Associated with the Use of Bioinoculants and Nanoparticles in Agriculture

While bioinoculants and nanoparticles have shown promise in improving agricultural practices, there are also some limitations associated with their use, i.e.:

- *Lack of standardization*: bioinoculants and nanoparticles are relatively new technologies, and there is a lack of standardization in terms of their production, application methods, and dosage. This makes it challenging to compare the effectiveness of different products and limits their widespread adoption [94]
- *Variable efficacy*: the efficacy of bioinoculants and nanoparticles can vary depending on various factors such as soil conditions, climate, crop type, and application methods. What works well in one context may not produce the same results in another, making it difficult to achieve consistent and predictable outcomes [22]
- *Environmental impact*: while bioinoculants and nanoparticles are generally considered environmentally friendly compared to traditional agricultural inputs, further research is still needed to understand their long-term impact on ecosystems. The potential accumulation of nanoparticles in soils and water bodies and their effects on nontarget organisms are areas of concern [37]
- *Cost considerations*: bioinoculants and nanoparticles can be relatively expensive compared to conventional agricultural inputs. The high production costs, limited availability, and lack of economies of scale contribute to their higher price tags, which may hinder their adoption, especially in resource-limited farming systems [38, 41]
- *Regulatory challenges*: the use of bioinoculants and nanoparticles in agriculture may face regulatory challenges due to their novel nature. Ensuring their safety for human health and the environment, establishing appropriate guidelines for their use, and obtaining necessary approvals can be time-consuming and complex, potentially slowing down their commercialization and adoption [42]
- *Limited understanding of long-term effects*: since bioinoculants and nanoparticles are relatively recent additions to agriculture, there is still a limited understanding of their long-term effects on soil health, microbial communities, and ecosystem dynamics. Further research is needed to assess the potential risks and benefits associated with their sustained use over extended periods [42]
- *Knowledge and awareness gaps*: farmers and agricultural practitioners may have limited knowledge and awareness about bioinoculants' and nanoparticles' proper use, benefits, and limitations. Education and outreach efforts are necessary to ensure that users have access to accurate information and understand the potential risks and challenges associated with these technologies [13]

It is important to note that ongoing research and development in the field of bioinoculants and nanoparticles is aimed at addressing many of these limitations. Continued scientific inquiry and practical field trials will contribute to a better understanding of their potential and enable informed decision-making regarding their use in agriculture.

## 10. Impact of Bioinoculants' and Nanoparticles' Agricultural Application on Global Food Security

The integration of bioinoculants and nanoparticles in agricultural practices has the potential to positively impact global food security in several ways [9, 11, 35].

- *Enhanced nutrient availability*: bioinoculants, such as beneficial microorganisms (e.g., bacteria and fungi), can improve nutrient availability in the soil by facilitating nutrient cycling, fixing atmospheric nitrogen, and solubilizing minerals. Nanoparticles can also enhance nutrient uptake by acting as carriers or slow-release systems for fertilizers. This increased nutrient availability can improve crop productivity, leading to higher yields and enhanced food production [9]
- *Disease and pest management*: bioinoculants can play a crucial role in the biological control of plant diseases and pests. Certain microorganisms can suppress the growth of pathogens or parasitic organisms, thereby reducing crop losses. Nanoparticles can also be used for targeted delivery of bioinoculants or for developing nanopesticides with enhanced efficiency and reduced environmental impact [11, 35]
- *Improved soil health and sustainability*: bioinoculants improve soil health by promoting beneficial microbial communities, enhancing soil structure, and reducing the need for chemical inputs [96]. Nanoparticles can aid in soil remediation and restoration efforts by helping to reduce soil erosion, increase water-holding capacity, and mitigate the adverse effects of pollutants. Healthy soils are essential for long-term agricultural productivity and sustainability [103]
- *Water management*: bioinoculants and nanoparticles can assist in efficient water management in agriculture. Certain microorganisms can enhance water uptake by plants and improve drought tolerance [21]. Nanoparticles, such as hydrogels, can act as water-absorbing materials that retain moisture in the soil, reducing irrigation requirements and water stress on crops [73]
- *Sustainable agriculture practices*: the integration of bioinoculants and nanoparticles aligns with the principles of sustainable agriculture. By reducing the reliance on synthetic fertilizers and pesticides, these practices can contribute to environmentally friendly and economically viable farming systems [5, 17]. This can have long-term benefits for global food security by preserving natural resources, minimizing negative impacts on ecosystems, and supporting resilient agricultural production [41]

While the integration of bioinoculants and nanoparticles shows great promise, it is important to note that their implementation should be supported by rigorous research, regulatory frameworks, and proper training to ensure safe and responsible use. Additionally, socioeconomic factors, access to technology, and knowledge dissemination should be considered to ensure equitable adoption across diverse agricultural systems and regions, thus maximizing the potential impact on global food security.

## 11. Current Challenges and Future Prospects for the Widespread Adoption of Bioinoculants and Nanoparticles in Sustainable Agriculture

The use of bioinoculants and nanoparticles in sustainable agriculture presents several challenges and future prospects for widespread adoption [5, 15, 34, 42, 61]:

- *Limited awareness and knowledge*: one of the primary challenges is the limited awareness and understanding of bioinoculants and nanoparticles among farmers and agricultural practitioners. Educating farmers about the benefits and applications of these technologies is crucial for their adoption [37]
- *Cost-effectiveness*: the cost of bioinoculants and nanoparticles can be a limiting factor for their widespread use. Making these technologies economically viable and accessible to small-scale farmers is important to encourage adoption [38, 41]
- *Standardization and quality control*: ensuring consistent quality and effectiveness of bioinoculants and nanoparticles is essential. Developing standardized production and quality control protocols is necessary to maintain reliability and build user trust [27, 42]
- *Regulatory framework*: bioinoculants and nanoparticles' regulation and approval process can be complex and time-consuming. Establishing clear regulatory frameworks and guidelines for their registration and commercialization is crucial for widespread adoption [42]
- *Long-term efficacy and environmental impact*: it is important to assess bioinoculants and nanoparticles' long-term efficacy and potential environmental impact. Research and monitoring are needed to evaluate their effectiveness, safety, and potential ecological consequences

### 11.1. Future Prospects

- *Sustainable farming practices*: bioinoculants and nanoparticles have the potential to contribute to sustainable farming practices by reducing the use of synthetic fertilizers, pesticides, and water. Their adoption can enhance soil health, nutrient availability, and pest management [10, 15, 20]
- *Increased crop productivity*: these technologies can improve crop productivity by promoting plant growth, enhancing nutrient uptake, and mitigating biotic and abiotic stresses. This can lead to higher yields and improved food security [67]
- *Environmental benefits*: bioinoculants and nanoparticles offer the opportunity to minimize the environmental impact of agriculture. They can reduce nutrient runoff, soil erosion, and chemical pollution, supporting ecosystem health and biodiversity conservation [39]
- *Precision agriculture*: integrating bioinoculants and nanoparticles with precision agriculture technologies, such as remote sensing and data analytics, can enable targeted application and optimize resource use. This can result in more efficient and sustainable agricultural practices [38, 40, 64]
- *Research and innovation*: continued research and development in bioinoculants and nanoparticles will lead to the discovery of new applications, improved formulations, and enhanced understanding of their mechanisms of action [20, 32, 67]. This will further drive their adoption and effectiveness in agriculture

## 12. Conclusion

The potential of utilizing PGPB instead of pesticides in agricultural settings is widely acknowledged as a promising and environmentally friendly alternative. However, to properly use this information in the field, one must first understand the specific plant-microbe interactions and investigate novel ways. Nanotechnology is an effective technique for overcoming challenges connected with microbial biofertilizers, which may be used in various contexts. Since a variety of nanomaterials have demonstrated favorable impacts, whether on plant growth promotion bacteria or the growth of a range of plant varieties, these nanomaterials are excellent choices for usage in biofertilizer formulations. Integrating nanomaterials containing beneficial bacteria in plants could increase the survivability and tolerance of microorganisms in the natural atmosphere, improving agricultural sustainability and plant development. However, different NPs have different effects on bacterial metabolism, and there are worries about the potentially dangerous repercussions of nanoparticles on the environment, the safety of food, and human health; thus, it is necessary to examine these effects. To determine the efficacy of this intricate system on the molecular level, it will be essential to conduct more experiments involving plant-growth-promoting bacteria, NP, and plants. The kind of bacteria and their physiology, physicochemical properties of nanomaterials, plant variety, and ecological relationships are all possible factors in the occurrence of this phenomenon. Most of the studies included in this review were conducted in controlled environments; hence, natural biotic and abiotic interactions in the field were not considered. For this reason, an in vivo experiment must be carried out to understand and validate the prior studies. Using nanomaterials in conjunction with PGPBs has the potential to boost yields in agricultural systems, which would help meet the growing need for food worldwide. Considering this, careful risk assessment techniques must be used to examine potential danger.

## Figures and Tables

**Table 1:** Regulatory effects of nanoparticles on plant gene expression [23–25].

Nanoparticle material	Effect on gene expression	Type of stress	Host plant
Silver	Upregulation of EIX and PAL genes	—	Tomato (*Solanum lycopersicum*)
	Upregulation of 438 genes	—	Arabidopsis (*Arabidopsis thaliana*)
	Upregulation of GSTU12, GR, GS, and PCS genes	—	
	Downregulation of 81 genes Upregulation of 286 genes	—	
	Upregulation of CHS and PAL genes	—	Black cumin (*Nigella sativa*)

Iron	Downregulation of OsHMA2, OsHMA3, and OsLCT1 genes	Cd and drought stress	Rice (*Oryza sativa*)

Copper	MVK gene downregulation, miR159 upregulation	—	Black pepper (*Piper nigrum*)

Silicon	Upregulation of B8AZZ8, Fo2, B8BF84, B8B. B8A9F5, and A2WZ30 genes	Biotic stress	Rice (*Oryza sativa*)
	Downregulation of RBOH1, APX2, ERF3, MPAK2, MAPK3, and DDF2 genes Upregulation of AREB, CRK1, TAS14, and NCED3 genes	Salinity	Tomato (*Solanum lycopersicum*)
	Upregulation of LSi2 and LSi1 genes	Salinity	Rice (*Oryza sativa*)

**Table 2:** Nanobiofertilizer's role in diverse plants under controlled and stressful conditions [16, 42–45].

Host plant	Stress environment	Formulation of nanobiofertilizer	Plant responses
*Phaseolus vulgaris* L.	Normal	Biofertilizer (*Rhizobium*) + organic fertilizer + ZnNPs	Increased biomass leaf area, plant height, and leaf number Increased pod yield, nutritional absorption, carbohydrate, and protein content
*Triticum aestivum* and *Cicer arietinum* L.	Biological stress	Biofertilizer (*Paenibacillus polymyxa*) + acylated homoserine-coated Fe-carbon nanofibres	Increased chlorophyll content, biomass, and length Pathogen-resistance development
*Solanum tuberosum* L.	Normal	Biofertilizer (nitroxin) + AgNPs	Increased plant tuber count, diameter, and weight
*Zea mays*	Normal	Biofertilizer (*Bacillus* spp.) + nanozeolite	Increased plant length, protein, and chlorophyll content
*Zea mays*	Drought stress	Biofertilizer + nanochelated B and Zn	Increases in chlorophyll content, ear length, ear diameter, leaf area, and relative water content among other morphological and physiological improvements Improved yield parameters
*Triticum aestivum*	Cd stress	Biofertilizer (composted biochar farmyard manure) + ZnO NPs	Increased plant photosynthetic pigments, antioxidants, biomass, and yield
*Triticum aestivum*	Stress and nonstress conditions	Biofertilizer (*Azotobacter*, *Pseudomonas*, and *Azospirillum*) + oxide NPs of Fe-Zn	Elevated levels of proline, soluble sugars, and enzyme activity Improved productivity by 88% under drought environment relative to the control group
*Triticum secale*	Cadmium stress	Biofertilizer (*Azotobacter chroococcum*, *A. caulinodans*, and *Azospirillum brasilense*) + TiNPs	Increased grain production, relative water, chlorophyll content, and grain weight Lowered Cd levels in leaves and seeds
*Sorghum bicolor* (L.) Moench.	Normal	Biofertilizer (*Azotobacter*) + nanofertilizer	Increased chlorophyll, carotenoid, and carbohydrate content
*Brassica juncea* L.	Heavy metal stress	Biofertilizer (compost and biochar) + zero-valent FeNPs	Total carbon, phosphorus, total nitrogen, and pH increased The growth and height of plants are improved

**Table 3:** The effect of nanoparticles and nanofertilizers on plant growth in the presence of adverse environmental conditions [45, 47, 50, 51, 89–92].

Plant	NPs	Impact of nanoparticle/nanobiofertilizer on plant	Amount applied in range	Inoculation approach used
Barley (*Hordeum vulgare* L.)	*n*CeO_2_	Enhanced plant productivity and higher concentrations of Ce in the grains, and increased levels of Al, Mn, Zn, Fe, K, P, Ca, as well as amino acids and fatty acids.	0–500 mg kg^−1^ soil	Soil
Wheat (*Triticum aestivum* L.)		Compared to typical plants, the plant was more fit and productive overall; nevertheless, while Ce uptake in the roots increased, there was no change in the seeds, hull, or leaves.	0–500 mg kg^−1^ soil	Soil
Wheat (*Triticum aestivum* L.)		Antioxidant enzyme activity is increased despite decreased photosynthetic pigments and seed protein. Plant biomass and productivity show no significant effect.	0–400 mg kg^−1^ soil	Soil
Cucumber (*Cucumis sativus* L.)		The pattern of carbohydrates has altered, but the level of starch has shown no change. Increased globulin concentration and decreased glutelin level.	400 mg kg^−1^ soil	Soil
Cilantro (*Coriandrum sativum* L.)		The roots had higher levels, CAT, and Ce in the stem.	0–500 mg kg^−1^ soil	Soil
Tomato (*Solanum lycopersicum* L.)	*n*CuO	Increased SOD levels, CAT, ABTS, vitamin C, and lycopene while decreasing GPX and APX activity. Elevated tomato fruit copper accumulation.	50–500 ppm (particle size 50 nm)	Foliar
Tomato (*Solanum lycopersicum* L.)		Enhanced fruit quality, production, and plant growth and development. Increased antioxidant and lycopene capacity.	0.02–10 ppm	Soil
Cucumber (*Cucumis sativus* L.)		ROS production was increased, as were phenolic compounds, amino acids, antioxidant enzyme systems, and citric acid levels.	10–20 ppm	Hydroponic
Cucumber (*Cucumis sativus* L.)		Fruit metabolites differed from those of control plants. Organic, amino, and fatty acids as well as sugars were improved.	40 nm (particle size)	Soil
Tomato (*Solanum lycopersicum* Mill.)		Improved biomass and growth of plant characteristics. Enzymatic activity, leaf gas exchange responses, and upregulated photosynthetic pigments.	10–100 mM	Soil
Onion (*Allium cepa* L.)	*n*CuO, *n*Al_2_O_3_, and *n*TiO_2_	Influenced mitotic index. Onion roots have higher ROS activity. An increase in the enzymatic activity of CAT and SOD was seen with all of the given NPs.	0–2000 *μ*g mL^−1^	Petri plate
Kidney bean (*Phaseolus vulgaris* L.)	*n*Cu/kinetin	Ca, Mn, and P levels of nutrients and chlorophyll content were decreased, whereas root Cu accumulation increased.	50, 100 mg kg^−1^ soil	Soil
Tomato (*Solanum lycopersicum* L.)	*n*Cu–chitosan	Enhanced stomatal conductance, plant performance, production, leaf CAT, and fruit lycopene levels.	0.3–0.015 M	Soil
Maize (*Zea mays* L.)	*n*Cu, *n*Fe, and *n*Co (metal NPs)	Enhanced SOD frequency, timing, enzymatic activity, early development, and metabolism in plant leaves to boost stress resistance.	3–5 ppm	Soil irrigation
Maize (*Zea mays* L.)	*n*SiO_2_	Increased biomass, nutrient uptake, thickness of cell wall, Si uptake, and germination rate (%).	20–40 nm	Hydroponic
Soybean (*Glycine max* L.)		Decreased plant root and leaf epidermis and pericycle Hg uptake, as well as the harmful effects on plant performance. Boost enzymatic reactions and leaf gas exchange.	30–50 nm (particle size)	Soil
Peregrina (*Jatropha integerrima*)		Increased growth characteristics and biochemical profile were observed.	1–2 mM	Foliar
Mahaleb (*Prunus mahaleb* L.)		When plants were pretreated with NPs at maximum treatment concentrations and improved nutritional level, i.e., N, P, and K content, improved photosynthetic performance was less affected by stress.	10–100 ppm	Soil irrigation
Faba bean (*Vicia faba* L.)		Increased productivity, plant size, seed quality, leaf biomass, germination rate, as well as the condition of the nutritional elements Na, Ca, K, P, and N.	1–3 mM	Soil
Cucumber (*Cucumis sativus* L.)		Overall improvement over control plants in terms of plant height, leaf count, area expansion, biomass, fruit weights, and quality.	15–120 ppm	Foliar
Strawberry (*Fragaria × ananassa*)		Plant stems now contain significantly more nutrition content for, e.g., Mn, Fe, Mg, Ca, K, and Si than before, while Cu and Zn levels remained the same.	20–80 ppm	Foliar and soil irrigation
Sugarcane (*Saccharum officinarum* L.)		Increased chlorophyll content, PS II apparatus, Fv/Fm variables, and photosynthetic efficiency under cold stress.	300 ppm	Foliar
Barley (*Hordeum vulgare* L.)		Significantly improved plant development, antioxidative enzyme activity, osmolytes, chlorophyll content, metabolite profile, and leaf gas exchanges.	12–250 ppm	Soil
Wheat (*Triticum aestivum* L.)		Reduces the damage that UV radiation causes to plants.	10 *μ*M	Hydroponic
Marigold (*Tagetes erecta* L.)		Improved biometrics and physiological, biochemical, and floral characteristics, such as fresh and dry flower mass, length of flowering, and time until first bud initiation.	100–600 ppm	Soil and foliar
—	Biogenic amorphous silica (bASi)	Increases the soil's ability to store water (SWHC). Increased bASi levels, increased soil water availability, and decreased water stress potential.	1–15%	Soil
Soybean (*Glycine max* L.)	*n*Fe_2_O_3_	Increased seed weight and leaf biomass compared to typical plants.	0.25–1 M	Foliar
Peanut (*Arachis hypogaea* L.)		Enhanced plant production, root shape, and growth characteristics. Increased levels of plant hormones, Fe absorption, enzymatic activity, Chl index, and photosynthetic pigments.	2–1000 ppm	Soil
Tomato (*Solanum lycopersicum* L.)		Enhanced seed germination, morphological characteristics, Fe uptake, and dry weight compared to control plants.	50–800 ppm	Hydroponic
Cucumber (*Cucumis sativus* L.)	*n*TiO_2_	CAT, APx, and enhanced leaf greenness were all decreased. TiO_2_ was applied, raising Kand P levels.	0–750 mg kg^−1^ soil	Soil
Barley (*Hordeum vulgare* L.)		When compared to untreated and treated plants, applied NPs were observed to promote plant performance by increasing germination (%).	500–1000 mg kg^−1^ soil	Soil
Tomato (*Solanum lycopersicum* L.)		Improved mineral absorption and accumulation by plants.	0–1000 mg kg^−1^ soil	Soil
Spinach (*Spinacia oleracea* L.)		Enhanced PS II oxygen-evolving rate (OER) and electron transport rate (ETR), enzymatic responses, and decreased ROS level.	0.25%	—
Wheat (*Triticum vulgare* L.)		No notable effects on the performance of the plant. As NP levels rose, leaf photosynthetic pigments decreased. Increased absorption and storage of nutrients, with the exception of the K level.	5–40 ppm	Hydroponic
Tomato (*Solanum lycopersicum* L.) and mung bean (*Vigna radiata* L.)	*n*TiO_2_-activated carbon composite	In tomato and mung bean, the right NP concentrations can speed up seed germination and shorten the germination time.	0–500 ppm	Foliar
Cucumber (*Cucumis sativus* L.)	*n*Fe_3_O_4_	Improved SOD and POD levels as well as plant growth, development, and yield. In order to solve issues with food security and safety, applied NPs improve/balance adequate nutrition management.	50–2000 ppm	Hydroponic
Barley (*Hordeum vulgare* L.)		Increase the number of chloroplasts, total soluble protein, photosynthetic pigments, and biomass attributes in plants. The excessive dose of NPs had no harmful effects. Excessive NP application decreased CAT and H_2_O_2_ activity, and changes were discovered in the genes responsible for photosynthetic plant leaves.	125–1000 ppm	Hydroponic
Chili (*Capsicum annuum* L.)	*n*Fe	The development of plants was found to benefit from low doses of *n*Fe. Improved grana stacking and chloroplast functional capability. High doses of FeNPs have been proven to harm plants and may halt the dispersion of the nutrient Fe.	0.002–2 mM L^−1^	Foliar
Tomato (*Solanum lycopersicum* L.)	*n*Ag	Improved root morphology, germination rate (%), and plant yield. A few genes were identified to have downregulated expression, including CRK1, MAPK2, P5CS, and AREB, which were found to have increased expression (TAS14, DDF2, and ZFHD1).	0.05–2.5 ppm	Seed
Tomato (*Solanum lycopersicum* Mill.)		The fruit's qualities and plant performance were improved by the use of NPs.	10–40 ppm	Soil irrigation
Soybean (*Glycine max* (L.) Mell.)		Hampered plant growth and N_2_ fixation.	31.2–62.5 mg kg^−1^ soil	Soil
Maize (*Zea mays* L.)	*n*ZnO	Enhanced physiological and metabolic processes under high pH treatment. Maximum growth characteristics.	150–300 ppm	Foliar
Mung bean (*Vigna radiata* L.)		Improved nutrient uptake, growth, and germination rate.	10–100 ppm	Petri plate
Tomato (*Solanum lycopersicum* Mill.)		Plants' negative impacts were lessened by ZnO NPs. A lower dose was preferable to a higher one. Different cultivars showed varying levels of stress tolerance.	15–30 ppm	Tissue culture
Maize (*Zea mays* L.)		Improved grain Zn accumulation, seed germination rate, seedling vigor index, biomass, and productivity.	50–2000 ppm	Foliar
Peanut (*Arachis hypogaea* L.)		Increased agricultural output, photosynthetic content, morphological characteristics, and general plant performance.	0–1000 ppm	Soil irrigation
Sweet basil (*Ocimum basilicum* L.)		Enhanced vegetative development, productivity of essential oils, biomass, and Zn content buildup.	1000 ppm	Foliar
Peanut (*Arachis hypogaea* L.)		Plant length, biomass, and the quantity and weight of pods are examples of morphological, yielding, and biochemical properties.	100–500 ppm	Soil
Sorghum (*Sorghum bicolor* L.)		Enhanced grain nutrient profile, uptake of N and K elements, improved plant performance and yield component, and NUE in comparison to typical plants.	6 mg kg^−1^ soil	Soil and foliar
Wheat (*Triticum durum*)	*n*Zn–chitosan	Increased Zn buildup in the crops grown on Zn-deficient agricultural soil.	20 mg g^−1^ soil (*w*/*w*)	Soil and foliar
Wheat (*Triticum aestivum* L.)	*n*Chitosan-NPK	Improved nutritional status, yield, and growth compared to typical plants.	500, 60, and 400 ppm (N, P, and K); 10, 25, and 100%	Foliar
Barley (*Hordeum vulgare* L.)	Chitosan	Improved the growth parameters, biochemical processes, yield, and leaf chlorophyll index.	30–90 ppm	Soil and foliar
Thale cress (*Arabidopsis thaliana* L.)	*n*Au	Increased growth, free radical scavenging, and seed germination (%) rates. Possible strategy to improve plant seed output.	10–80 *μ*g mL^−1^	Foliar
Cucumber (*Cucumis sativus* L.)	Mn_3_O_4_	Significantly improved plant biomass, photosynthetic activity, chlorophyll content, and plant development. Increased endogenous antioxidant defense systems.	1–5 mg plant^−1^	Foliar

## Data Availability

No data are available.
